# Patients’ sense of security from clinical factors in Iran: a cross-sectional study

**DOI:** 10.1186/s12913-024-10677-x

**Published:** 2024-02-28

**Authors:** Roya Malekzadeh, Ghasem Abedi, Arash Ziapour, Murat Yıldırım, Ehsan Abedini

**Affiliations:** 1 Department of Health Services Management, Faculty of Health, Health Sciences Research Center, Mazandaran University of Medical Sciences, Sari, Iran; 2 Cardiovascular Research Center, Health Institute, Imam-Ali Hospital, Kermanshah University of Medical Sciences, Kermanshah, Iran; 3 Department of Psychology, Faculty of Science and Letters, Agri Ibrahim Cecen University, Ağrı, Turkey; 4 Graduate Studies and Research, Lebanese American University, Beirut, Lebanon; 5 Mazandaran University of Medical Sciences, Sari, Iran

**Keywords:** Sense, Security, Patient, Nursing factors, Medical factors

## Abstract

**Background:**

One of the clinical responsibilities and goals of hospitals is to provide patients with comfort and security. The present study aims to assess patients’ sense of security among patients in Iranian hospitals.

**Methods:**

The present research employed a cross-sectional design. The sample consisted of 830 patients visiting public, private, and social security hospitals in Mazandaran in the North of Iran. The required data were collected using a questionnaire developed by the researcher of this study.This questionnaire consisted of 4 dimensions:nursing, medical, advanced facilities and patient rights. The participants were selected using a proportional stratified random sampling method. Exploratory factor analysis, confirmatory factor analysis, descriptive statistics, and ANOVA were used for data analysis using SPSS version 22.

**Results:**

The mean scores of patients’ sense of security in social security, private, and public hospitals were 4.16 ± 0.89, 3.78 ± 0.67, and 3.60 ± 0.89, respectively. Medical factors with a mean and standard deviation of 3.92 ± 0.76, advanced facilities and equipment with 3.89 ± 0.89, nursing factors with 3.87 ± 0.73, and patient rights with 3.71 ± 0.90 were the highest to the lowest scores, respectively. The results showed that the type of hospital significantly affected the mean dimensions of security (*p* < 0.05).

**Conclusions:**

The study revealed variations in the sense of securityacross the sampled hospitals. Particularly, the sense of security attributed to the patient rights factors was lower than other factors. Therefore, to enhance the sense of security for patients, it is recommended to focus on staff training and fostering a culturethat emphasizes obtaining informed consent, demonstrating respect for the patient, and introducing the medical team to the patient before initiating any treatment.

**Supplementary Information:**

The online version contains supplementary material available at 10.1186/s12913-024-10677-x.

## Introduction

Patients’ sense of security is one of the first responsibilities of healthcare providers. The patients feel safe are treated better, are discharged faster, and their medical expenses will be lower [1]. Security background is as long as human history and dates back to the formation of society and community concepts [2]. Its decline can lower humanpeace of mind and replace it with agitation, anxiety, and unrest [3]. Security presence is as important as the sense of security. It can be claimed that the sense of security is more important than its presence because one’s reactions are contingent upon the rate of one’s perception of security. Patients’ sense of security has been highlighted recurrently in palliative and medical care. The existing literature shows that the sense of security significantly affects the provision of soothing care and helps patients improve. For example, patients who trust the staff and their competence to provide services will recover faster [4]. Providing comfort to patients can prevent adverse physiological effects and have a positive effect on their physical and mental status and can lead to a faster recovery and discharge [5].

According to Milberg Anna’s study, there is a statistically significant relationship between a patient’s sense of security and demographic characteristics (e.g. sex and age), diagnosis type (e.g. cancer), health-associated quality of life, the perceived support from family and friends, self-efficiency, stress level, the intensity of symptoms of the disease, and coping with diseases [6, 7]. The “presence of a nurse” causes the patient to feel mentally safe. Most patients stated that the constant presence of nurses at their beds gives them a sense of security and peace of mind that will lead to comfort and improvement of health [8, 9]. Patient comfort resources in a hospital setting include four dimensions: “feeling kind and close relationship with nurses, understanding the constant presence and monitoring of the nurse, supporting family and peer patients, and being in a safe and comfortable hospital environment” [10]. Juybari et al.(2006) stated that features such as “self-awareness, availability of nurses, supervision and retention by nurses, caring behaviour of medical staff, and professional competence of staff” put patients’ mind at rest. On the contrary, the lack of physical, psychosocial, environmental and financial comfort makes them uncomfortable [5].

Krevers and Milberg (2015) developed an instrument for the sense of security in care—relatives’ evaluation, in which patients’ awareness of the sense of security was questioned and the sense of security in care was considered very important to maintain and improve the quality of care. The results of the study showed that the three axes of care interaction, identity, and mastery were associated with patients’ sense of security [11]. Also, Nadighara et al., (2016) designed a questionnaire on patients’ sense of security in four dimensions of clinical services, hoteling factors, access and Supportive– psychological [12], Based on this questionnaire, only the patients’ sense of security in the fild of hoteling factors was evaluated. The results reported by Abedi et al., (2015) in exploring the sense of security in a sample of hospitals using a Hoteling Service Approach showed that the sense of security developed through providing hoteling services among the patients visiting the social security hospital was stronger than those visiting private and public hospitals. Moreover, the sense of security induced by health and sanitation services such as cleaning the toilets, hospitalization wards, and hospital campuses was higher than other factors [13]. The study conducted by Abedi et al., (2019), which explored the effect of patients’ security rights and medical errors on patients’ sense of security showed that in social security, public and private hospitals, patients’ sense of security is related to three factors, namely patients’ safety, medical errors and patient’s rights, and this correlation is stronger in social security hospitals than the others [14].

In light of the aforementioned issues, every organization needs to establish trust and a sense of security for its customers and clients. Hospitals, as complicated organizations, also need to recognize relaxation requirements due to their importance in treatment. Therefore, the medical staff should have sufficient knowledge about patient’s needs, how to satisfy them, and the requirements to adequately satisfy them [15, 16]. When patients are comforted, they cope with stressful conditions and recover more quickly [8].

The review of the literature showed that despite the design of the security feeling questionnaire, clinical services in patients were not investigated and so far no internal and external studies have been conducted regarding the evaluation of clinical factors including medical and nursing factors and advanced facilities on the patient’s sense of security. Therefore, this study aimed to assess patients’ sense of security from clinical factors in Iran. It is hoped that the results of the present study can provide appropriate information about the patient’s sense of security to the managers of the health system and lead to making appropriate decisions at the micro and macro levels to provide better services and create favourable conditions for the treatment of patients and reduce treatment costs and improve health services.

## Methods

### Study design

A cross-sectional design was used in the current study. The participants were inpatients visiting public, private, and social security hospitals in Mazandaran (Northern Iran) in 2019. Social security hospitals are affiliated with social security organizations for specific target populations. The participants(inpatients) were selected using a proportional stratified random sampling method. To cover the estimated sample size, in the first step, three hospitals were randomly selected out of three stratums (public hospitals (*N* = 25), private hospitals (*N* = 9) and social security hospitals (*N* = 5). Then, inpatients in three hospitals (public, private and social security) were selected using a systematic random method. The sample size in three hospitals (public, private and social security) was estimated using Cochran`s formula with a standard error of 0.05 (d = 0.05) and a type I error of 0.03 (α = 0.03). The sample size was in public (*n* = 342), private (*n* = 245) and social security (*n* = 254) hospitals. The total sample size was 841 inpatients. A researcher-made questionnaire was used to collect the data. This questionnaire was distributed in Persian. This instrument was designed by studying the relevant articles and literature on the sense of security and according to the standards provided by the World Health Organization and the Ministry of Health. Exploratory factor analysis and confirmatory factor analysis were run among 841 patients and the data were collected and analyzed. Its dimensions were determined precisely after an exploratory factor analysis, which included 4 factors such as the nursing factors (5 questions), medical factors (5questions), patient rights (5questions), and advanced facilities (5questions) and a total of 20 questions.

The results of the confirmatory factor analysis showed satisfactory goodness of fitness indices of the model with values of 0.079 for RMSEA, 2.11 for X2 / df, 0.90 for GFI, 0.92 for AGFI, and 0.073 for RMR, respectively. These prove the good fitness of the measured model. The validity of the measurement instrument was substantiated by ten experts. The reliability was determined by Cronbach’s Alpha test of internal consistency. The reliability of different sections of the scale, such as the nursing factors, medical factors, patient rights and advanced facilities was 0.93, 0.94, 0.89 and 0.89, respectively. Thus, the reliability was substantiated. The total Cronbach’s Alpha was 0.86. The rating of the questionnaire was on a Likert scale. The options of the scale ranged from very low to very high (very low = 1, low = 2, average = 3, high = 4, and very high = 5). The minimum score for each question was 1 and the maximum score was 5. The average score in each question and factor was calculated. According to the Likert scoring scale, it was obtained from the sum of the scores of each question divided by the number of participants. The exclusion criteria were outpatients, patients with clinical unconsciousness and children under 12 years of age. Finally, out of 841 questionnaires that were distributed, 830 questionnaires were filled and used in the present study. The data were analyzed through descriptive statistics and analysis of variance (ANOVA) test in SPSS 22.0.

## Results

The results showed that 340 participants (40.9%) visited public hospitals, 241 (29.0%) the private and 249 (30.1%) the Social Security hospitals. Independent T Test showed that there is not any difference between male and female groups base on sense of security, while ANOVA test illustrated that the sense of security is different two other variables age, Educational level, and Patients job (Sig. < 0.05) (Table 1).

**Table 1:** Demographic variables

Demographic variable		n	%	P-value
Sex	female	421	50.7	0.77
	male	409	50.3	
Age	< 20 years	48	5.8	0.021
	20–30 years	234	28.2	
	30–40 years	296	35.7	
	40–50 years	166	20.0	
	> 50 years	86	10.3	
Educational level	Illiterate	32	3.8	0.045
	Below school diploma	90	10.9	
	school diploma	220	26.5	
	Bachelor’s degree	391	47.1	
	Master’s degree and higher	97	11.7	
Patients job	Unemployed	41	4.9	0.003
	Employed	352	42.4	
	Retired	179	21.5	
	Housewife	164	19.8	
	University student/ student	94	11.4	

To determine the sense of security of inpatients, the mean score in each question and dimension was calculated (1 ≤ mean ≤ 5). The mean scores of security sense among the patients visiting Social Security, Private, and Public hospitals were 4.16 ± 0.89, 3.78 ± 0.67, and 3.60 ± 0.89, respectively. Nursing factors in social security hospitals (4.12 ± 0.72) were more than in private and public hospitals. The same results were obtained for medical factors, patient rights, and advanced facilities. Medical factors with a mean and standard deviation of 3.92 ± 0.76, advanced facilities and equipment 3.89 ± 089, nursing factors 3.87 ± 0.73 and patient rights 3.71 ± 0.90 were, respectively, the highest and the lowest scores of conditions of establishing a sense of security. The hospital type affected the score of the dimensions of the sense of security, and individuals who had visited Social Security hospitals had higher mean scores (Nursing factors, medical factors, patient rights and advanced facilities) than those who had visited private and public hospitals (Table 2).

**Table 2:** Descriptive statistics of dimensions of the sense of security

Clinical Factors	Hospitals	Mean	Std. Deviation
Nursing factors	Public	3.67	0.88
	Private	3.82	0.56
	Social Security	4.12	0.72
	Total	3.87	0.73
Medical factors	Public	3.64	0.77
	Private	3.89	0.61
	Social Security	4.25	0.91
	Total	3.92	0.76
Patient rights	Public	3.50	0.93
	Private	3.52	0.75
	Social Security	4.11	1.02
	Total	3.71	0.90
Advanced facilities	Public	3.61	1.00
	Private	3.91	0.78
	Social Security	4.15	0.91
	Total	3.89	0.89
sense of security	Public	3.60	0.89
	Private	3.78	0.67
	Social Security	4.16	0.89
	Total	3.84	0.81

The results showed that among the nursing factors, nurses’ technical skills (4.78 ± 1.56) had the highest score and patient education (3.51 ± 0.70) had the lowest score. Among the medical factors, establishing proper communication with the patient (4.18 ± 0.97) had the highest score and timely presence to visit patients (3.89 ± 0.88) had the lowest score. Among the patient rights, keeping secrets and confidentiality (4.10 ± 1.1)7 had the highest score and informed consent (3.56 ± 0.86) had the lowest score. Among the advanced facilities, the existence of complementary sections such as special sections ICU and CCU (4.05 ± 0.97) had the highest score and the variety of hospital specialties (3.61 ± 0.74) had the lowest score (Table 3).

**Table 3:** Descriptive statistics of security sense items

Clinical Factors	Actions	Mean	Std. Deviation
Nursing factors	Patient education	3.51	0.70
	Timely attendance at the patient’s bedside	4.21	1.04
	Appropriate behavior	3.75	0.78
	Technical skills of the nurse	4.78	1.56
	Patient supervision by the nurse	3.69	0.96
Medical factors	Accuracy in diagnosing the disease	4.11	1.20
	Establishing proper communication with the patient	4.18	0.97
	Appropriate behavior	3.97	0.90
	Timely presence to visit patients	3.89	0.88
	Answer patient questions	3.91	1.01
Patient rights	Informed consent of the patient	3.56	0.86
	Keeping secrets and confidentiality	4.10	1.17
	Respect for the patient’s privacy	3.85	0.87
	Respect for the patient’s religious beliefs	3.74	0.82
	Advice on decision-making in treatment matters	3.98	0.90
Advanced facilities	The existence of complementary sections such as special sections ICU and CCU	4.05	0.97
	The existence of paraclinical imaging sections such as MRI and CT scan	3.87	1.08
	Providing comprehensive paraclinical laboratory services	3.93	0.81
	Providing medical services with new methods	3.89	0.96
	Variety of hospital specialities	3.61	0.74

Additionally, ANOVA test was used to compare the mean scores of quantitative variables across three hospitals. As seen in Table 4, the F-values showed that the type of hospital affectedthe mean dimensions of the sense of security, significantly (*P* < 0.05). To identify specific differences, we conducted post hoc analysis. The results of Tukey post hoc test indicated that the nursing factors were significantly different across three paired hospitals (i.e. public-private, public-social security, and private-social security). Medical factors were significantly different in three paired hospitals (i.e. public-private, public-social security, and private-social security). Similarly, advanced facilities differed significantly across three paired hospitals (i.e. public-private, public-social security, and private-social security). Patient rights differed significantly just in two paired hospitals (i.e. public-social security and private-social security) (*P* < 0.05)(Table 5).

**Table 4:** Sense of security in different hospitals

Effect		Value	F	Hypothesis df	Error df	P-value	Partial Eta Squared
Intercept	Pillai’s Trace	0.849	5867.012b	6.000	896.000	0.000	0.675
	Wilks’ Lambda	0.034	5867.012b	6.000	897.000	0.000	0.675
	Hotelling’s Trace	36.578	5867.012b	6.000	895.000	0.000	0.675
	Roy’s Largest Root	36.576	5867.012b	6.000	895.000	0.000	0.675
Hospital	Pillai’s Trace	0.169	15.213	12.000	1914.000	0.000	0.822
	Wilks’ Lambda	0.718	15.213b	12.000	1913.000	0.000	0.822
	Hotelling’s Trace	0.187	15.241	12.000	1913.000	0.000	0.822
	Roy’s Largest Root	0.144	19.897c	6.000	1912.000	0.000	0.822

**Table 5:** Dimensions of the sense of security in different hospitals

sense of security dimension	(I) - (J)	(I) - (K)	(J)– (K)
Nursing factors	-0.15 ± 0.32*	-0.45 ± 0.16**	-0.30 ± 0.16*
Medical factors	-0.25 ± 0.16*	-0.61 ± 0.14**	-0.36 ± 0.30*
Patient right	-0.02 ± 0.18	-0.61 ± 0.09**	-0.59 ± 0.27**
Advanced facilities	-0.30 ± 0.22*	-0.54 ± 0.09*	-0.24 ± 0.13*

(I) public; (J) private; (K) Social Security

*P < 0.05; **P < 0.01

## Discussion

This study aimed to determine the effective factors in the sense of security among patients visiting public, private, and social security hospitals in Mazandaran city in Iran. The findings revealed that in social security, public and private hospitals, patients’ sense of security is related to four factors, including nursing factors, medical factors, patient rights and advanced facilities and this correlation is more obvious in social security hospitals than the private and public ones. The findings reported by Amiri et al., (2022), showed that the rate of sense of security among patients in public hospitals was significantly lower than that of the private sector [17]. The findings by Rashidian et al. (2011) in Tehran [18]and Javadi et al.(2011), in Isfahan, a major city in central Iran, [19] showed that private hospitals are more accountable than state ones, which is in line with the present findings. However, the results of another study by Abedi et al., (2015), on the sense of security in hoteling showed that the sense of security in hoteling in private hospitals is more than state and social security ones [13]. In a study by Ebrahimpour et al., (2014), in Mashhad, no significant difference was found in response between state and private hospitals [20]. One reason for this variance is that no similar study has been conducted and the existing literature deals with the analysis of responses by centres. One reason for the difference in the sense of security based on hospital ownership in the present study could be that patients visited social security hospitals for free, and also the average payment to staff is higher than public hospitals, and it is private, which can make you feel safe.

The results showed that the sense of security was more influenced by medical factors than by other factors. In this regard, Rostami et al., (2018), figured out that the doctor-patient relationship was the most important factor affecting an individual’s satisfaction with medical-health systems. This relationshipnot only impacts patient satisfaction but also influences adherence to medical instructions and the recovery process [21].. Milberg et al., (2014), found a significant associations between patient satisfaction and patient-doctor relationship [22].

In the present study, concerning the development of a sense of security, nursing factors were ranked third among various clinical factors. The results of another study by Sheikh Beiklou et al., (2012), showed a significant relationship between nurses’ relationship with other medical staff and patient security [23]. The findings reported by Amiri et al., (2022), also highlighted this finding [24]. Zani et al., (2014), indicated that communication can contribute to an effective, therapeutic action that does not only heal the body but also brings comfort to the spirit [25]. The results of another study by Nadighara et al.(2016), showed if the nurse prioritized her paperwork and did not talk to the patient while providing care, the patient would feel unwell [12]. Kwame et al., (2022), stated that nurse-patient communication improves patient satisfaction with care outcomes and empowers both individuals and families [26]. Vitale et al., (2021) showed that good communication between nurses and patients is essential for the successful outcome of individualized nursing care for each patient [26]. Good communication also is not only based on nurses’ physical abilities but also on education and experience.

In the present study, patient rights as a factor that leads to patients’ sense of security ranked last. Although all studies focusing on this issue have highlighted the importance of patient rights, this factor has not gained priority. Most studies emphasized on the importance of patient rights and dignity [21–25, 27]. The study conducted by Abedi et al., (2019), which explored the effect of patients’ security rights and medical errors on patients’ sense of security showed that in social security, public and private hospitals, patients’ sense of security is related to patients’ rights [14]. However, each of these studies highlighted one or more different aspects of patient rights. Hajibabaee et al., (2021), found that the nurses’ awareness about patient rights was optimal [28]. But Jouzi Arkavazi et al., (2013) reported that only 3.8% of patients were aware of patient rights [29]. The results of a study by Keshtkaran et al., (2017), showed that only 22% of the nursing staff indicated that the patient was allowed to use the device and 8% indicated respect for the patient when the patient’s name was not mentioned [30]. The lack of respect for the patient and the observance of patient privacy from the patient’s point of view have been shown in several studies [31, 32]. The results from earlier studies by Begley et al., (2018) in the Czech Republic and Ishola et al., (2017) in Nigeria revealed that many women experienced disrespectful care during child delivery [29, 30]. In contrast, the level of respectful care in East and Southern Africa ranged between 62% and67% [31]. It seems that the low level of awareness of respect for patient rights is one reason for paying little attention to the effect of this factor on the patient’s sense of security.

The present study addressed comprehensive influential factors that contribute to patients’ sense of security. These factors must be addressed to enhance the sense of security among patients in the clinical setting. Nursing managers can adopt multiple strategies to enhance patients’ sense of security to promote patient safety and nurses’ practice. Additionally, hospital managers can use medical factors, nursing factors, patient rights and advanced facilities to improve patient’s sense of security. One limitation of the study was the time and place of conduction, so the results cannot be generalized to other hospitals. Design and psychometrics of patients’ sense of security measurement instruments are the strengths of this study.

## Conclusion

To conclude, the present findings showed nursing and medical factors, patient rights and advanced facilities have significant effects on patients’ sense of security, simultaneously. Considering the above-mentioned, those factors which are related to medicine (e.g., accuracy in diagnosing the disease, communication with the patient, appropriate behaviour, regular presence to visit patients and answer his/her questions) have the greatest impact and those which are related to patient rights (informed consent of the patient, keeping secrets and confidentiality, patient’s privacy, patient’s religious beliefs and advice on decision-making in treatment matters) have the least impact on patients’ sense of security in public, social and private hospitals. Therefore, emphasis on the human basic rights in healthcare and treatment, especially respect for the status of a patient as a human being, is important. The present study reveals the effect of observing patients’ rights on safety and comfort in medical centres. Respect for the dignity of the patient is an effective step to increase patient satisfaction with the services provided by the medical staff. It is important to participate in patients’ decision-making and preserve their rights to improve treatment, reduce hospitalization rates and cater for cost-effective treatment, due to the harmful effects of efficacy and other harmful effects on the body.

### Electronic Supplementary Material

Below is the link to the electronic supplementary material.

Supplementary Material 1

Supplementary Material 2

## Data Availability

The datasets generated and/or analysed during the current study are not publicly available due to consent not being obtained from participants for this purpose but are available from the corresponding author on reasonable request.

## Abbreviations

PSS: Patients’ sense of security
PM: Precision medicine
CF: clinical Factors
CSS: cross-sectional study

## References

[1] Kwame A, Petrucka PM (2022). Universal healthcare coverage, patients’ rights, and nurse-patient communication: a critical review of the evidence. BMC Nurs.

[2] Kashkoli SA, Zarei E, Daneshkohan A, Khodakarim S (2017). Hospital responsiveness and its effect on overall patient satisfaction: a cross-sectional study in Iran. Int J Health Care Qual Assur.

[3] Norouzi F, Follady Sephr S (2010). Sense of social security and social factors affecting women 1529 years old in Tehran. Persian J Rahbord.

[4] Igarashi A, Miyashita M, Morita T, Akizuki N, Akiyama M, Shirahige Y (2012). A scale for measuring feelings of support and security regarding cancer care in a region of Japan: a potential new endpoint of cancer care. J Pain Symptom Manag.

[5] Jouybari L, Oskouie F, Ahmadi F (2006). Comfort of hospitalized patients: a missed concept. Iran J Nurs.

[6] Milberg A, Friedrichsen M, Jakobsson M, Nilsson E-C, Niskala B, Olsson M (2014). Patients’ sense of security during palliative care—what are the influencing factors?. J Pain Symptom Manag.

[7] Moradi F, Tourani S, Ziapour A, Abbas J, Hematti M, Moghadam EJ (2021). Emotional intelligence and quality of life in elderly diabetic patients. Quart Community Health Educ.

[8] Molazem Z, Ahmadi F, Mohammadi E, Bolandparvaz S (2011). Improvement in the nursing care quality in general surgery wards: Iranian nurses’ perceptions. Scandinavian J Caring Sci.

[9] Lebni JY, Toghroli R, Abbas J, Kianipour N, NeJhaddadgar N, Salahshoor MR (2021). Nurses’ work-related quality of life and its influencing demographic factors at a public hospital in Western Iran: a cross-sectional study. Quart Community Health Educ.

[10] Shafi PV, Mohammadi E, Ahmadi F (2013). Experiences of open heart surgery patients from admission to discharge: a qualitative study. Iran J Crit Care Nurs.

[11] Krevers B, Milberg A (2015). The sense of security in Care—Relatives’ evaluation instrument: its development and presentation. J Pain Symptom Manag.

[12] Nadighara AA, Abedi G, Abedi E, Rostami F (2016). Designing and validating a scale to measure the sense of security in hospitalized patients. J Mazandaran Uni Med Sci.

[13] Abedi G, Abedini E, Mahmmodi G, Mlakzadeh M, Malakzadeh R (2015). Investigating the level of patients’ security in the selected hospital of Mazandaran province using hoteling service approach. Int J Pharm Technol.

[14] Abedi G, Mahmoodi G, Malekzadeh R, Khodaei Z, Siraneh Belete Y, Hasanpoor E (2019). Impact of patients’ safety rights and medical errors on the patients’ security feeling: a cross-sectional study. J Hum Rights Healthc.

[15] Renauer BC (2007). Reducing fear of crime: citizen, police, or government responsibility?. Police Quart.

[16] Mohammadi M, Ziapoor A, Mahboubi M, Faroukhi A, Amani N, Hydarpour F (2014). Performance evaluation of hospitals under supervision of kermanshah medical sciences using pabonlasoty diagram of a five-year period (2008–2012). Life Sci J.

[17] Amiri M, Khosravi A, Raei M (2022). Patients’ sense of security in Shahroud (Northeast Iran) hospitals. Open Public Health J.

[18] Rashidian A, Majdzadeh R, Pourreza A, Pourmalek F, Arab M, Mohammad K (2011). Assessing health system responsiveness: a ousehold survey in 17th district of tehran. Iran Red Crescent Me J.

[19] Javadi M, Karimi S, Raiesi A, Yaghoubi M, Kaveh K (2011). Comparison of patients’ and nurses’ viewpoints about responsiveness among a sample from public and private hospitals of Isfahan. Iran J Nurs Midwifery Res.

[20] Ebrahimpour F, Shahrokhi A, Ghodousi A (2014). Patients’ safety and nurses’ medication administration errors. Iran J Forensic Med.

[21] Farmahini Farahani M, Kashani nia Z, Hosaini MA, Biglarian A (2007). The effect of nurses communication s ills training on patient satisfaction with nurse-patient intraction. Iran Nurs Res.

[22] Heading GM, Sinclair S, ishop J (2009). Cancer patient satisfaction survey.

[23] Hemmati -Maslakpak M, Sheikhbaglu M, Baghaie R (2014). Relationship between the communication skill of nurse - patient with patient safety in the critical care units. J Clin Nurs Midwifery.

[24] Amerion A, Ebrahimnia M, Karimi Zarchi A, Sh T, Zaboli R, Rafati H (2009). Inpatient and outpatient satisfaction of a military hospital. J Mil Med.

[25] Zani AV, Marcon SS, Tonete VLP, de Lima Parada CMG (2014). Communicative process in the emergency department between nursing staff and patients: social representations. Online Brazilian J Nurs.

[26] Kourkouta L, Papathanasiou IV (2014). Communication in nursing practice. Materia Socio-Med.

[27] Rao JK, Weinberger M, Kroenke K (2000). Visit-specific expectations and patient-centered outcomes: a literature review. Arc Family Med.

[28] Bahrani N, Hooshmand A, Joolaee S, Mehrdad N (2006). Nurses’ information and their view points about patient’s rights and practical facilitators in clinics. Hayat.

[29] Jouzi Arkavazi HA, Abbasi M, Delpishe A, Mannati R, Shahmir L (2013). Organizational factors related to the rights of patients by nurses and patients in hospitals affiliated with the University of Ilam. J Med Ethics.

[30] Keshtkaran A, Taft V, Keshtkaran V, Heidari A, Shahmohammadi J, Dehbozorgi M (2017). Client tribute plan and patient satisfaction in Shiraz hospitals. Payavard Salamat.

[31] Hasan Tehrani T, Seyed Bagher Maddah S, Fallahi-Khoshknab M, Mohammadi Shahboulaghi F, Ebadi A (2018). Perception hospitalized patients from respect for privacy. Iran Nurs Res.

[32] Ingstad K, Pedersen MK, Uhrenfeldt L, Pedersen PU (2023). Patients’ expectations of and experiences with psychosocial care needs in perioperative nursing: a descriptive study. BMC Nurs.

