# Supplementary material for: Patients’ sense of security from clinical factors in Iran: a cross-sectional study
Source: BMC Health Serv Res. 2024 Feb 28;24:259. doi: 10.1186/s12913-024-10677-x (PMC10902943; doi:10.1186/s12913-024-10677-x)
Supplement: Supplementary file 1 — Supplementary Material 1 [file 12913_2024_10677_MOESM1_ESM.doc]

**Patients’ sense of security questionnaire from clinical Factors**

Hello and respect

This questionnaire was compiled in order to carry out a research project titled "**Patients’ sense of security from clinical Factors in Iran: A cross-sectional study**". Please help us to use your valuable opinions by completing this questionnaire. It is necessary to remember that there is no need to mention your name and family name in completing the questionnaire, and your statements will be confidential and will be used exclusively in the research. Thank you for your help**.**

What extent did each of the following factors in relation to the clinical services provided in the hospital affect your sense of security in this hospital?

| Very low  1 | Low  2 | average 3 | high  4 | very high  5 | Question | Factors |  |
| --- | --- | --- | --- | --- | --- | --- | --- |
|  |  |  |  |  | Patient education by the nurse | Nursing | 1 |
|  |  |  |  |  | Timely attendance of the nurse at the patient's bedside |  | 2 |
|  |  |  |  |  | Appropriate behavior of the nurse with the patient |  | 3 |
|  |  |  |  |  | Technical skill of the nurse |  | 4 |
|  |  |  |  |  | Patient supervision by the nurse |  | 5 |
|  |  |  |  |  | Aaccuracy in diagnosing the disease by the doctor | Medical | 6 |
|  |  |  |  |  | Establishing proper communication between the doctor and the patient |  | 7 |
|  |  |  |  |  | Aappropriate behavior of the doctor with the patient |  | 8 |
|  |  |  |  |  | Timely presence of the doctor to visit patients |  | 9 |
|  |  |  |  |  | Answer patient questions by the doctor |  | 10 |
|  |  |  |  |  | Obtaining the conscious consent of the patient | Patient right | 11 |
|  |  |  |  |  | keeping patient secrets and confidentiality |  | 12 |
|  |  |  |  |  | Respect for the patient's privacy |  | 13 |
|  |  |  |  |  | Respect for the patient's religious beliefs |  | 14 |
|  |  |  |  |  | Consultation with the patient on decision-making in treatment matters |  | 15 |
|  |  |  |  |  | Existence of complementary sections such as special sections ICU and CCU | Facilities | 16 |
|  |  |  |  |  | Existence of paraclinical imaging sections such as MRI and CT scan |  | 17 |
|  |  |  |  |  | Providing comprehensive paraclinical laboratory services |  | 18 |
|  |  |  |  |  | Provide medical services with new methods |  | 19 |
|  |  |  |  |  | Existence of various hospital specialties |  | 20 |
