# Supplementary material for: Patients’ sense of security from clinical factors in Iran: a cross-sectional study
Source: BMC Health Serv Res. 2024 Feb 28;24:259. doi: 10.1186/s12913-024-10677-x (PMC10902943; doi:10.1186/s12913-024-10677-x)
Supplement: Supplementary file 2 — Supplementary Material 2 [file 12913_2024_10677_MOESM2_ESM.docx]

**Patients’ sense of security questionnaire from clinical Factors**

A researcher-made questionnaire was used to collect the data. This questionnaire was distributed in Persian. This instrument was designed by studying the relevant articles and literature on the sense of security and according to the standards provided by the World Health Organization and the Ministry of Health. Exploratory factor analysis and confirmatory factor analysis were run among 841 patients and the data were collected and analyzed. Its dimensions were determined precisely after an exploratory factor analysis, which included 4 factors such as the nursing factors (5questions), medical factors (5questions), patient rights (5questions), and advanced facilities (5questions) and a total of 20 questions.

The results of the confirmatory factor analysis showed that the fitness indices of the model such as RMSEA, X2 / df, GFI, AGFI, and RMR were 0.079, 2.11, 0.90, 0.92, and 0.073 respectively. Theseprove the good fitness of the measured model. The validity of the measurement instrument was substantiated by ten experts. Thereliability was determined by Cronbach’s Alpha test of internal consistency. The reliability of different sections of the scale, such as the nursing factors, medical factors, patient rights and advanced facilities was 0.93, 0.94, 0.89 and 0.89, respectively. Thus, thereliability was substantiated. The total Cronbach’s Alpha was 0.86.
